# Roles of Mesenchymal Stem Cells in Spinal Cord Injury

**DOI:** 10.1155/2017/5251313

**Published:** 2017-05-28

**Authors:** Jing Qu, Huanxiang Zhang

**Affiliations:** Department of Cell Biology, Jiangsu Key Laboratory of Stem Cell Research, Medical College of Soochow University, Ren Ai Road 199, Suzhou Industrial Park, Suzhou 215123, China

## Abstract

Spinal cord injury (SCI) represents one of the most complicated and heterogeneous pathological processes of central nervous system (CNS) impairments, which is still beyond functional regeneration. Transplantation of mesenchymal stem cells (MSCs) has been shown to promote the repair of the injured spinal cord tissues in animal models, and therefore, there is much interest in the clinical use of these cells. However, many questions which are essential to improve the therapy effects remain unanswered. For instance, the functional roles and related molecular regulatory mechanisms of MSCs in vivo are not yet completely determined. It is important for transplanted cells to migrate into the injured tissue, to survive and undergo neural differentiation, or to play neural protection roles by various mechanisms after SCI. In this review, we will focus on some of the recent knowledge about the biological behavior and function of MSCs in SCI. Meanwhile, we highlight the function of biomaterials to direct the behavior of MSCs based on our series of work on silk fibroin biomaterials and attempt to emphasize combinational strategies such as tissue engineering for functional improvement of SCI.

## 1. Introduction

Spinal cord injury (SCI) usually results in severe neural dysfunction below the injury site. Moreover, mammals are unable to regenerate their spinal cords after injury which can lead to lifelong disability and loss of independence. After a primary damage of spinal cord tissue by a direct mechanical force, a series of secondary events involving various pathological responses accelerate the tremendous cell loss, release of cytotoxic factors, and cystic cavitation [1, 2]. Furthermore, excessive extracellular matrices produced by activated astrocytes, called glial scarring, together with the hostile microenvironment, severely inhibit cell migration and axonal regrowth [3]. Although many experimental and clinical studies have been tested, it still lacks effective treatment until now [4–6]. The neuropathological outcome of SCI is complicated, and therefore, several challenging objectives, such as decreasing neural cell death, reducing scarring and cavitation, regaining healthy neural cells, and stimulating functional axonal regeneration, remolding the injury niche should be taken into consideration [7–11].

Numerous studies have demonstrated that stem cells might provide a source of neural cells as well as exerting neuroprotective effects after SCI. Among them, mesenchymal stem cells (MSCs) emerged as one of the most promising types of stem cells due to a favorable ethical profile and better safety [12]. The present data revealed that recovery after MSC implantation therapy is comparatively low possibly because of uncertain neural plasticity and limited capacity for the axonal regeneration of MSCs in the spinal cord [13, 14]. The therapeutic application of MSCs in SCI is still in its infancy. It is of considerable interest as to how stem cells respond to the local environment and play functional roles in vivo, which will provide important information for improving the therapy effects and designing better therapeutic strategies.

## 2. The Biological Behavior of MSCs In Vivo

### 2.1. Migration of MSCs

A few points need to be taken into account to obtain more effective stem cell therapy outcomes. For instance, it is important for transplanted cells to arrive and migrate into the injured spinal cord tissue after intravenous infusion. It has been demonstrated that MSC homing toward injured tissue is not an efficient process; very few cells reach the injury site [15]. Some of the transplanted cells were trapped into the lung and other organs while many cells were sacrificed during the journey [16]. And only a small percentage of cells were verified to have high homing ability since the transplanted MSCs are always mixed cell populations. There are experimental data that support that MSCs possess high migratory potential and higher ability to help neural regeneration. In this case, it is believed that the insufficient number of migratory cells will partly account for the decreased number of transplanted MSCs and further decreased the cell therapy effects.

On the other hand, it is also crucial for MSCs to migrate and integrate into the host spinal cord tissue after cells are injected into a lesion, or close to a lesion area. It is not surprising that people may feel confused: Why do cells need to migrate if they are already in the lesion area? We noticed that cells would die quickly if they stayed in the injection site by in situ MSC transplantation after SCI. Actually, MSCs were observed to be migrating away from the injection site in the first 1 hour after cell transplantation. By 7 days, the cells had migrated across the injury site to form a cellular scaffold, suggesting migration toward the injury sites [17]. Also, some cells with neuronal marker expression were observed in the injured and surrounding tissues after MSC transplantation [18]. However, the engraftment potential of MSCs was low which was verified by many experiments. Indeed, MSCs delivered via injection largely remained restricted to the lesion site and were not seen to contact significant amounts of the host spinal cord tissue. The numbers of the engrafted cells are dramatically decreased after transplantation by either in situ injection or intravenous infusion [19]. It was reported that there were small numbers, even less than 0.001% to 0.002%, of the transplanted MSCs left, and few functional neurons were detected after cell transplantation [20–23].

There are studies showing that the migratory and homing capacities of MSCs are closely related to their engraftment and regeneration ability. After transplantation, grafted MSCs, which possess higher migratory ability, exhibited greater survival at the periphery of the lesion. Consistently, the motor functions of the rats that had received these grafts improved significantly [21]. These data establish the fact that better recovery of damaged tissues via stem cell therapy demands sufficient recruitment of transplanted cells to the target tissue. Interestingly, it was shown that the migratory behaviors of Drosophila stem cells are closely related to their regeneration ability too. For example, hindgut stem cells of Drosophila would begin to differentiate and replace the damaged cells and tissues as long as they migrate to arrive at the right place, which is controlled by the Wnt and Hh signaling pathways [24, 25]. The mechanisms of MSC migration and homing were extensively investigated too. Studies have demonstrated that MSCs strongly respond to inflammatory or chemotactic stimuli released from injured tissues including chemokines and various growth factors like vascular endothelial growth factor (VEGF), hepatocyte growth factor (HGF), and SDF-1*α*/CXCR4 axis [26, 27]. Studies indicate that MSCs with enhanced migratory ability to the lesion site following SCI enhance the antiapoptotic effects by upregulating the expression of stromal cell-derived factor-1 (SDF-1)/CXC chemokine receptor 4 (CXCR4) axis. In one investigation, impaired expression of CXCR4 and cell engraftment was observed in populations of bone marrow MSCs [28]. Consistently, the SDF-1*α*/CXCR4 axis enhances cell migration toward injured tissues and promotes recovery after SCI by mediating bone marrow MSCs [29, 30]. Besides, substance P that acts as a neurotransmitter was able to mobilize MSCs from the bone marrow and subsequently enter into the impaired tissues [31]. Granulocyte-colony-stimulating factor (G-CSF) was also known to promote mobilization of MSCs to the injured tissue [32]. Our recent study demonstrates that calcitonin gene-related peptide (CGRP) is one of the key factors that regulate the homing of transplanted MSCs to sites of SCI [21]. It looks like there are many factors that regulate the migratory behavior of MSCs. Usually, MSC cultures are initiated with a heterogeneous, poorly defined cell population. It is unknown which MSC populations are expanded and how this process affects homing capacity. There is evidence that only a small percentage of MSCs are able to migrate toward different chemotactic stimuli. We found that MSCs in varying neural differentiation states display different chemotactic responses to HGF. In addition, the phosphorylation levels of PI3K/AKT or MAPK signaling were closely related to the migration efficiency of MSCs [33]. Other authors reported that a population of CD34^−^ adult bone marrow-derived stem cells do not express functional CXCR4, or only a small proportion of MSCs express functionally active CXCR4 [34]. Probably, different mechanisms are involved to induce cell migration for different subpopulations of mixed MSC cultures. Precise “homing” mechanism of the transplanted cells to the lesion site is still largely unknown, which is of great interest for future study.

### 2.2. Differentiation of MSCs

Morphological studies showed that neuronal and oligodendroglia cell protein markers are expressed in transplanted MSCs after SCI [35]. For example, small amounts of fluorescent-tagged MSCs can be found in the blood vessels in the area of SCI where they can differentiate into NSE-positive neurons, indicating that MSCs can migrate into the injured area and differentiate into neuron-like cells. In another study, expression of *β*III-tubulin at the injury site was verified indicating the potential for functional regeneration. Moreover, grafted MSC can differentiate into myelin-forming cells in the completely transected rat spinal cord [36]. However, it was reported that transplanted cells were identified adjacent to neurons and astrocytes after SCI, but no cells were seen to be labeled with any neural markers at any time [37]. Although some groups have found neuronal differentiation of MSCs in vivo, the survival number of grafted and differentiated neurons were too small to be considered to contribute to functional recovery after SCI [38, 39]. Moreover, these cells, sometimes, do not show specific neuronal electrophysiological properties [40]. Indeed, controversial opinions are coexisted regarding the neural differentiation capacity of MSCs in vivo. Many experimental data support the opinion that the ability of MSCs to secrete soluble factors or vesicles rather than engrafting and transdifferentiating plays an important role in SCI repair [41–43].

### 2.3. Gene Therapy to Increase Nerve Regeneration of Transplanted MSCs after SCI

Efforts were made to increase the regeneration efficacy of MSC therapy for SCI. A previous study has shown that MSCs expressing the Shh transgene could increase cell survival after transplantation [44]. At day 28 after treatment, more MSCs were present in the injured tissue in the Shh-MSC group than in the MSC group. Furthermore, the transplanted cells expressing Shh exhibit enhanced functional recovery of neurological function after SCI in rats. Kumagai et al. verified that transplantation of MSCs expressing MNTS1, a multineurotrophin that binds TrkA, TrkB, and TrkC and p75NTR receptors, led to recovery of sensory function, promoting axonal growth after SCI [45]. Similarly, a series of studies indicated that NT3 or other neurotrophin gene-transfected MSCs are an effective approach to improve nerve regeneration and functional recovery after SCI [46–52].

Recently, central roles for microRNAs (miRNAs) as core regulators of gene expression during central nervous system (CNS) pathologies were revealed by many studies [53, 54]. It has been shown that overexpression of miRNA-21 dramatically downregulates expressions of caspase-3, Fas ligand, and programmed cell death (PDCD4), improves the survival of intact motor neurons, and exerts neuroprotective effects on spinal cords against ischemia-reperfusion injury [55]. More recently, both in vitro and in vivo studies found that miR-133b promotes neurite outgrowth and improve functional recovery after SCI while the detailed mechanisms need to be evaluated further [56]. The polypyrimidine tract-binding proteins (PTBPs) are one of the important RNA-binding protein family members, which are thought to be involved in cell-specific alternative splicing. PTBP1 and its brain-specific homologue polypyrimidine tract-binding protein 2 (PTBP2) regulate neural precursor cell differentiation [57]. Experimental data demonstrated that specific miRNA, like miR-124, could promote the productivity of neurogenic cells (NSE-positive cells) by increasing PTBP2 expression of stem cells. Moreover, neurogenic cells derived from miR-12-overexpressed stem cells successfully participate in neural restoration after SCI [58, 59]. These findings provide important regulatory roles of miRNAs in response to CNS damage and encourage novel therapy targeting miRNAs and their target genes for SCI in the future.

## 3. Function of MSC Transplantation after SCI

### 3.1. Animal Model

MSC implantation exerts a therapeutic effect on experimental SCI animal models, which is supported by evidence of functional recovery [12]. However, the precise function of MSC transplantation has not been clarified until now. It is expected that after cell transplantation, MSCs would be able to differentiate into specialized neuronal and glial cell lineages. The neural differentiation ratio is low and these kinds of neurons did not show specific neuronal electrophysiological properties sometimes. Although there are controversies, the present data support that the efficacy of MSCs is mainly based on paracrine and neuroprotection functions like secreting numerous growth factors and trophic factors rather than differentiation [42, 60–62].

In general, the function of MSC transplantation includes both structural and functional benefits. Recent data show that MSC transplantation prevented cavity formation due to SCI and resulted in subsequent motor recovery after SCI [63–65]. At the same time, MSC admission promotes recovery of bladder and hindlimb function after SCI in rats [66]. Matsushita et al. suggest that intravenously delivered MSCs have important effects on reducing blood spinal cord barrier leakage, which could contribute to their therapeutic efficacy too [67]. MSCs are immune-privileged cells that may cross human leukocyte antigen barriers to facilitate transplantation [41, 64, 68, 69]. In other studies, reduction of inflammatory infiltrates and decrease of cell apoptosis at the lesion epicenter of the spinal cord are observed after MSC transplantation [61, 70–73]. MSCs are able to reprogram macrophages from a proinflammatory M1 phenotype toward an anti-inflammatory M2 phenotype and also able to regulate immune response in the injured spinal cord to provide a permissive environment for axonal extension and functional recovery [74]. Proteomic analysis of the conditioned medium of MSCs reveals a novel set of inducers for anti-inflammatory M2-like macrophages, such as monocyte chemoattractant protein-1 (MCP-1) [75]. Depletion of MCP-1 from conditional medium decreases MSCs' abilities to induce M2 macrophages and recovery from SCI. Hence, the therapeutic effect of MSC transplantation is partly based on MSCs' paracrine function, such as their ability to secrete trophic factors. Besides MCP-1, nerve growth factor (NGF), brain-derived neurotrophic factor (BDNF), neurotrophin-3 (NT-3), and many other growth factors are also increased after MSC transplantation for SCI [42, 76].

Generally, most stem cell therapy studies have focused on the acute or subacute phase, while there are a limited number of studies evaluating treatment efficacy during the chronic phase of SCI. There are data indicating enhanced therapeutic effects of MSC transplantation at 9 days postinjury period rather than the transplantation immediately after injury. Indeed, subacute intraparenchymal grafting of syngeneic MSCs has only a minor effect on functional recovery [77]. The function of stem cell transplantation approach for SCI might be different depending on the different time phases [16]. For chronic SCI, MSCs were transplanted 8–10 weeks after the induction of SCI and an improved functional recovery and neural regeneration was verified [78, 79]. The systemic infusion of MSCs resulted in functional improvement, which is associated with structural changes, including stabilization of the blood-spinal cord barrier (BSCB), axonal sprouting/regeneration, and remyelination. However, anti-inflammation strategies would be needed to further improve the chronically injured spinal cord, which could be a challengeable mission of MSC transplantation for chronic SCI treatment.

### 3.2. Clinical Trials

Based on preclinical experiments in SCI animal model showing MSC transplantation in the improvement of functional recovery after SCI, a series of clinical trials were performed. These experiments showed that the grafting of such cells is safe and brings benefits for some patients by using different cell application methods and transplantation procedures [80]. Collectively, autologous MSC transplantation has been shown to be an overall safe and well-tolerated procedure. Intralesional transplantation of autologous MSCs in subjects with complete SCI is safe, is feasible, and may play some roles to promote neurological improvements [81, 82]. Consistently, an approach to personalized cell therapy in chronic SCI indicated that all patients experienced improvement, primarily in sensitivity and sphincter control, while intralesional motor activity, according to clinical and neurophysiological studies, obtained an improvement by more than 50% of the total 12 patients [83]. A case report indicated that MSC transplantation can partially promote recovery of deep sensory pathways as demonstrated by somatosensory evoked potential (SSEP) recording and alleviate neuropathic pain of a patient with traumatic complete cervical SCI [84].

However, this is not always the case. Recently, a study reported a clinical trial which made an attempt to track bone marrow-derived MSCs in a patient with a chronic cervical SCI. The results suggested that tagged bone marrow-derived stem cells were detected at the patient's cervical spinal cord with magnetic resonance imaging at 48 hours, which faded after two weeks, and then disappeared after one month. Unfortunately, no clinical improvement of the neurological function had occurred at the end of this study [85]. Similarly, there are also data indicating that there is no significant improvement in Basso, Beattie, and Bresnahan (BBB) score after MSC transplantation for SCI [86, 87]. Pal et al. reported that there is no effectiveness of the treatment involved after MSC injection for a of total 20 SCI patients during 1–3 years follow-up [88]. So, there is a common concern regarding the efficiency and reproducibility of the therapeutic use of MSCs for SCI patients. It is necessary to ensure the efficacy of MSCs as therapeutic agents for SCI before recommending clinical application of this treatment at this time.

Among various strategies for SCI treatment, it is generally accepted that stem cell transplantation is a good candidate approach leading to recovery of neural function [89, 90]. MSC transplantation shows some improvements in a varying degree of functional recovery after SCI. However, there are many concerns before MSC application into SCI patients extensively [1]. Until now, many neuroprotection roles of MSC transplantation were reported for SCI treatment. Here comes the question: which one or none of them plays a central role? The answer to this question is important for us to understand the repair mechanisms of stem cells, consequently ensuring the effectiveness of cell therapy and developing new strategies for SCI treatment. In general, MSCs from the bone marrow or other sources are mixed with different cell populations, which display complex antigen expression profiles. Thus, we have no idea about which specific cell population produces the best therapy effects. Moreover, MSC therapy only partly improves neurological function, which is not good enough when being applied to treat chronic SCI. Hence, MSC transplantation is not an effective and reliable therapy for SCI so far. More studies need to be done before massive clinical therapy is applied.

## 4. MSC and Tissue Engineering

Considering the complex environment and cell interactions within the spinal cord, a combination of stem cells with other treatment strategies, like application of biomaterials, might bring up better results [16, 91, 92]. Generally speaking, suitable biomaterials should have some special characteristics, such as biocompatibility, porosity and permeability for the diffusion of ions, nutrients, and waste products, and biodegradability. More importantly, biomaterials should have the capacity of mimicking the extracellular matrix (ECM) of CNS tissues, which provides a more permissive environment for cell survival, growth, migration, and differentiation [93]. Therefore, they are expected to provide an adequate environment for the regeneration of the injured tissues. Taking into account the well-known capacity of MSCs to secrete paracrine factors and the neural protection function when transplanted into spinal cord lesion models, their combination with a 3D matrix holds great promise to SCI repair [94–97].

Silks are naturally occurring polymers that have been used clinically as sutures for centuries. Silk fibroin in various formats has been shown to support cell adhesion, proliferation, and differentiation in vitro with a variety of cells and promote tissue repair in vivo. The work from our groups found that electrospun silk fibroin (SF) nanofibers support the adhesion and growth of neural cells. Interestingly, our data indicated that nanofibers could help neurons form the three-dimensional network by providing the supported substrate. At the same time, SF nanofibers promote neurite outgrowth and astrocyte migration [98]. Furthermore, we proposed that the diameter of biodegradable SF polymer could influence the growth behavior of cells in vitro. In conclusion, our in vitro data demonstrate that smaller diameter and aligned electrospun tussah silk fibroin represent valuable scaffolds for supporting and promoting growth and migration of stem cells, thus raising the possibility of manipulating SF scaffolds to enhance growth, homing, and therapeutic potential of stem cells in cellular therapy [99].

Besides natural biomaterials, biodegradable synthetic scaffolds have been used to support and improve the stem cell regenerative performance too. Hydrogels are particularly appealing for neural tissue repair because of their special physical properties such as being injected into the body in a localized and noninvasive manner [100, 101]. In a more recent study, a new agarose/carbomer-based hydrogel which combines different strategies to optimize MSC viability was evaluated. The study demonstrates that a combination of MSCs and biomimetic hydrogel is able to immunomodulate the proinflammatory environment in a SCI mouse model and promote a favorable regeneration environment in situ significantly [96]. This study presents the ability of a 3D ECM deposition to increase adherence and viability of loaded human MSCs.

**Figure 1:**
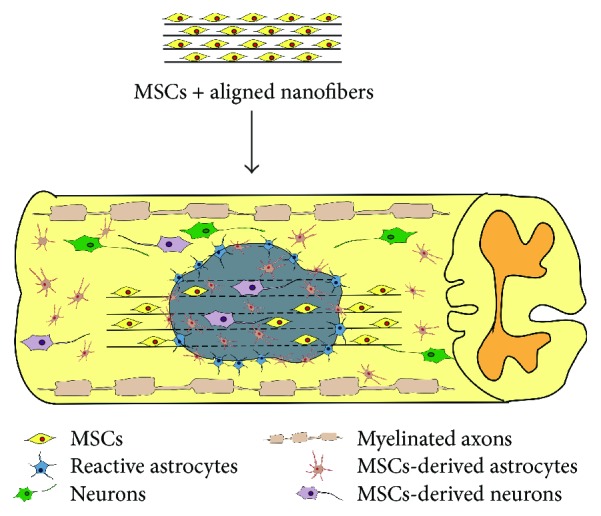
Biomaterials with different topographies have the capacity of mimicking the ECM of the CNS tissue and further influencing the growth behavior of transplanted stem cells. The aligned nanofibers were supposed to improve the migration and differentiation of cells after SCI.

As mentioned previously, the biological behavior of MSCs in vivo is closely related to their regeneration ability. For instance, the migratory and differentiation capacities of MSCs are closely related to their engraftment and regeneration ability. Therefore, one of the principal functions of nerve tissue-engineered scaffolds is to direct neural cell behavior such as growth, cell spreading, migration, and differentiation and to respond to the environment in a targeted implantable tissue. Hyatt et al. showed that MSCs delivered via scaffold formed longitudinally aligned layers growing over the spinal cord lesion site [102]. Host neurites within the spinal cord tissue were found to migrate into the graft. In addition, the layered architecture of the scaffold appeared to induce cell/tissue polarity and promote longitudinal growth of neurites within the graft [103]. Also, multichannel/laminin (LN) silk scaffolds could mediate cell migration, stimulate blood capillary formation, and promote axonal extension, suggesting a strong correlation between scaffold topography and growth behavior of stem cells [104]. More intensive studies are required for the investigation of the activities of stem cells after being combined with biomaterials to offer insights into the design and development of nerve tissue engineering scaffold especially for SCI (Figure 1) [105].

## 5. MSC-Derived Exosomes as a Promising Therapeutic Vesicle for SCI

Secretomes, also called extracellular vesicles, are several groups of secreted vesicles, which could be classified as exosomes, microvesicles (MVs), and apoptotic bodies. Exosomes (30–100 nm) can be distinguished from MVs (100–1000 nm) and apoptotic bodies (1000–5000 nm) according to their size, morphology, origin, composition, and density [106]. They are membrane-bound vesicles which are secreted naturally by many types of cells. Exosomes contain proteins, lipids, and various nucleic acids, including mRNAs, miRNAs, and long noncoding RNAs (lncRNAs) [107]. These exosomal RNAs can be taken up by distant cells and lead to the protein translation in the target cells. Thus, exosomes function as natural carriers of signal molecules and further act as physiological regulators of cell-to-cell communication. Recently, several studies indicate that lncRNAs in cancer exosomes can act as diagnostic and prognostic biomarkers [108, 109]. The discovery of their regulatory roles on distinct physiological or pathological conditions has brought increasing attention to exosomes.

MSC exosomes, like exosomes in general, carry exosome-associated markers such as Alix, tetraspanins (CD9, CD63, and CD81), and heat-shock proteins including Hsp60, Hsp70, and Hsp90. Besides, the other distinct composition of MSC exosomes depends on cell sources (which tissue MSCs were isolated from) and their physiological states [93]. There are around 857 unique gene products and more than 150 kinds of miRNAs expressed in MSC exosomes, suggesting that exosomal proteins and RNAs could form different functional RNA-protein complexes to perform diverse cellular responses [110].

Exosomes derived from MSCs may have a comparable therapeutic potential as cells themselves. Studies showed that exosomes derived from MSCs have therapeutic potential for many kinds of diseases [111]. For example, exosomes derived from MSCs exert protective effects on myocardial ischemia/reperfusion injury. MSC-derived exosomes can reverse the degeneration of neurons and astrocytes, as well as synaptic loss in hippocampus of diabetic mice [112]. Zhang et al. demonstrated that exosomes derived from MSCs can promote axonal growth of cortical neurons, indicating a potential therapeutic strategy to enhance axonal growth after CNS injury [113]. Moreover, MSC exosomes contribute to the improvement of impaired neurological functions, implying their potential clinical applications [114]. These results raise a possibility that exosomes derived from MSCs might be a promising therapeutic tool for SCI. However, there still lack direct experimental evidence that administration of cell-free exosomes generated from MSCs promotes axonal growth and improves neurological functions after SCI.

In general, MSCs represent the most promising source of exosomes for the neurotherapeutic applications. MSCs were found to produce large amounts of exosomes and could be used as the source to produce commercially sustainable production of exosomes. Exosomes are less immunogenic, more biocompatible and stable, compared to other existing viral or liposome-based gene delivery. It has been proposed that exosomes may cross the blood-brain barrier and enter into the CNS via intercellular junctions of endothelial cells. In addition, exosomes can be modified with genetic engineering, which will improve their therapeutic efficiency. These characteristics suggest that exosomes can be developed as an ideal vehicle for therapeutic delivery. However, exosomes contain a diverse array of signaling molecules with complicated functions, which could raise multiple safety issues. Therefore, it is critical for future studies to engineer exosome delivery systems containing high density of the defined therapeutic molecules, which target specific cells on the given situations.

## 6. Molecular Mechanisms after MSC Transplantation for SCI

It is well known that MSCs can produce various growth factors, neuroprotective cytokines and chemokines, including HGF, VEGF, fibroblast growth factor (FGF), BDNF, and NGF, which could indeed underlie functional benefits associated with MSC transplantation [115, 116]. Recent studies demonstrated that MSCs are an efficient source of HGF and suggest that the therapeutic effects of MSC transplantation are partly mediated by HGF secreted by these cells [117]. HGF blocked secretion of transforming growth factor-*β* (TGF-*β*) from activated astrocytes and prevented expression of specific chondroitin sulfate proteoglycan (CSPG) species. Transplantation of HGF-overexpressing MSCs markedly decreased Neurocan expression and glycosaminoglycan chain deposition around hemisection lesions in the spinal cord. Animals treated with HGF-MSCs showed increased axonal growth and improvement in functional recovery [118], which is consistent with the view that HGF have been identified as attractive signals for guidance of motor axons to the target tissue [119]. In addition, HGF has been reported to provide therapeutic effects in central nerve injury, such as the suppression of demyelination, apoptosis, and blood-brain barrier disruption, through the c-Met receptors that are upregulated after injury in rat neurons, oligodendrocytes, and astrocytes [120].

Besides growth factors which act as paracrine signaling, immunological cytokines are also involved in the process of stem cell therapy after SCI. For instance, transplantation of MSCs into a lesion spinal cord reduced the secretion of TNF*α*, IL-4, IL-1*β*, IL-2, IL-6, and IL-12 when compared to that of the saline-treated controls [121–123]. Particularly, implantation of MSCs prevents second-phase neuronal injury by suppressing lymphocyte and microglia effects and reduces the inflammatory reaction in the local environment after SCI [124]. These results indicate that neuronal survival after lesion might occur through cytokine release and immunomodulation followed by MSC administration.

Previous studies reveled that MSC implantation modulates glial scar formation after SCI. One of these reports concludes that MSC treatment after SCI upregulates matrix metalloproteinase- (MMP-) 2 levels and reduces the formation of the glial scar thereby creating an environment suitable for endogenous regeneration mechanisms [125]. In addition, it was shown that human MSCs deposit fibronectin (FN) following SCI, which is a well-known inducer of axonal growth, as well as a component of the extracellular matrix (ECM) [126]. Importantly, it has been shown that FN secreted by MSCs are essential for neurite elongation of neuronal differentiating MSCs as well as nerve fiber regeneration after SCI. Laminin is a well-known inducer of axonal growth too, as well a component of the ECM associated to neural progenitors. Laminin and TGF-*β* expression have also been increased in the injured spinal cord after MSC admission for SCI. The in vivo data suggest that laminin can be the paracrine factor mediating the proregenerative effects of MSCs in spinal cord injury [127].

Apoptosis-related pathways have been found involved in SCI after MSC transplantation. Recent findings suggest that caspase-3-mediated apoptosis on both neurons and oligodendrocytes following SCI was significantly downregulated by MSCs, which was regulated through stimulation of endogenous survival signaling pathways, PI3K/Akt, and the MAPK/ERK1/2-cascade [128]. Extracellular-adjusting protein kinases 1 and 2 (ERK1/2) are important intracellular signaling molecules that are members of the MAPK family. Consistently, Wang et al. showed that transplanting MSCs activates ERK1/2 in spinal cords of ischemia-reperfusion injury rats and improves nerve function [129]. At the same time, Bcl2 expression increased, whereas Bax expression decreased following stem cell transplantation. There are also data indicating that transplantation of MSCs for neurological disorders inhibited apoptosis and the protein expression of c-Jun N-terminal kinase and p38 as well triggered the phosphorylation of P-42/44 ERK1/2 [130]. However, it remains undetermined whether MAPK/ERK1/2-cascade participates in other mechanisms beyond inhibition of apoptosis, such as secretion of various neurotrophic factors that promote the regeneration or improving the axon regeneration microenvironment.

**Figure 2:**
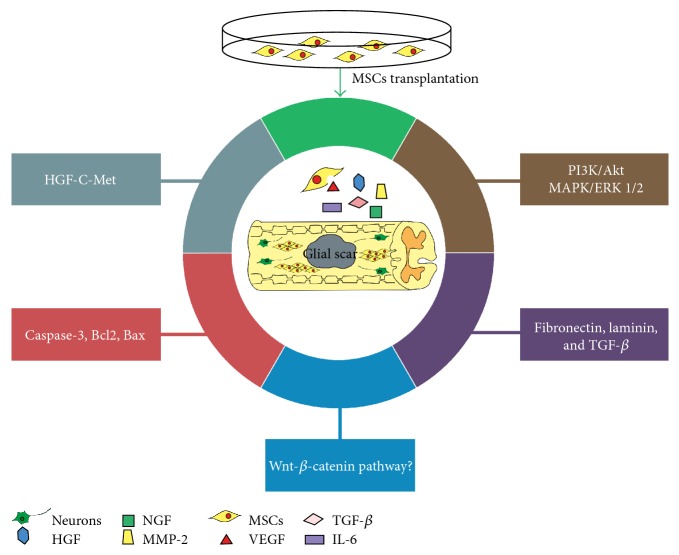
Regulatory molecular mechanisms involved in SCI after MSC transplantation.

It has been demonstrated that Wnt/*β*-catenin signaling plays a key role in promoting the differentiation of MSCs toward a neuronal fate. Wnt-7a enhanced neuronal differentiation in MSCs via both canonical and noncanonical signaling pathways [131]. Contusion spinal cord injury induced a time-dependent increase in Wnt expression from 6 hours until 28 days postinjury. Specially, after an initial decrease by 1 day, an increase in phosphorylation of the Wnt coreceptor, low-density lipoprotein receptor-related protein 6 (LRP6), and an increase in active *β*-catenin protein were shown, indicating that canonical Wnt signaling is active in the adult spinal cord and in cells around the wound epicenter after SCI [132]. There is some evidence that spinal radial glia, neural progenitors in zebrafish, exhibit canonical Wnt/*β*-catenin activity as they undergo neurogenesis following spinal cord transection [133]. Wnt/*β*-catenin signaling may promote axon regrowth either directly or through induction of secondary pathways in radial glia, suggesting important regulating roles in neural regeneration. In addition, overexpression of Dkk1b, an inhibitor of Wnt/*β*-catenin signaling, hampers locomotor recovery, axon regeneration, and glial bridge formation in the regenerating spinal cord of adult zebrafish. However, it is still undetermined in mammals that whether Wnt/*β*-catenin signaling is the activated response to SCI after MSC implantation, which might be explored in the near future (Figure 2).

## 7. Conclusions

MSCs are considered as the most promising sources for cellular therapies following SCI. The mechanisms underlying the biological behavior of MSCs and their complicated function in vivo are not fully understood, which is very important for improving the therapeutic effects and for designing better therapeutic strategies. A combination of MSCs with nerve tissue-engineered scaffolds can direct cell behavior such as growth, cell spreading, migration, and differentiation and respond to the local environment after SCI. More intensive studies are required for the investigation of the activities of cells after combined with biomaterials to offer insights into the design and development of nerve tissue-engineering scaffold for SCI. MSCs represent the most promising source of exosomes for the neurotherapeutic applications, and exosomes derived from MSCs may have a comparable therapeutic potential as cells themselves. Notably, MSCs respond to the local environment in multiple ways. MSCs produce various growth factors, neuroprotective cytokines and chemokines, reduce the inflammatory reaction by suppressing lymphocyte effects, modulate glial scar formation, downregulate Caspase-3 mediated apoptosis by activating ERK1/2-cascade, and so forth. In addition, Wnt/*β*-catenin signaling pathway might also play important regulatory roles for MSC behavior after SCI. In conclusion, it is of considerable interest to investigate the biological behavior and function of MSCs, especially after SCI treatment. The regulatory mechanisms directing MSC behavior in molecular details will undoubtedly provide valuable insights in improving the MSC-mediated therapy effects and designing better therapeutic strategies.
